# Genenames.org: the HGNC resources in 2023

**DOI:** 10.1093/nar/gkac888

**Published:** 2022-10-16

**Authors:** Ruth L Seal, Bryony Braschi, Kristian Gray, Tamsin E M Jones, Susan Tweedie, Liora Haim-Vilmovsky, Elspeth A Bruford

**Affiliations:** HUGO Gene Nomenclature Committee, European Molecular Biology Laboratory, European Bioinformatics Institute, Wellcome Genome Campus, Hinxton CB10 1SD, UK; Department of Haematology, University of Cambridge School of Clinical Medicine, Cambridge CB2 0PT, UK; HUGO Gene Nomenclature Committee, European Molecular Biology Laboratory, European Bioinformatics Institute, Wellcome Genome Campus, Hinxton CB10 1SD, UK; HUGO Gene Nomenclature Committee, European Molecular Biology Laboratory, European Bioinformatics Institute, Wellcome Genome Campus, Hinxton CB10 1SD, UK; Department of Haematology, University of Cambridge School of Clinical Medicine, Cambridge CB2 0PT, UK; HUGO Gene Nomenclature Committee, European Molecular Biology Laboratory, European Bioinformatics Institute, Wellcome Genome Campus, Hinxton CB10 1SD, UK; HUGO Gene Nomenclature Committee, European Molecular Biology Laboratory, European Bioinformatics Institute, Wellcome Genome Campus, Hinxton CB10 1SD, UK; HUGO Gene Nomenclature Committee, European Molecular Biology Laboratory, European Bioinformatics Institute, Wellcome Genome Campus, Hinxton CB10 1SD, UK; HUGO Gene Nomenclature Committee, European Molecular Biology Laboratory, European Bioinformatics Institute, Wellcome Genome Campus, Hinxton CB10 1SD, UK; Department of Haematology, University of Cambridge School of Clinical Medicine, Cambridge CB2 0PT, UK

## Abstract

The HUGO Gene Nomenclature Committee (HGNC) assigns unique symbols and names to human genes. The HGNC database (www.genenames.org) currently contains over 43 000 approved gene symbols, over 19 200 of which are assigned to protein-coding genes, 14 000 to pseudogenes and nearly 9000 to non-coding RNA genes. The public website, www.genenames.org, displays all approved nomenclature within Symbol Reports that contain data curated by HGNC nomenclature advisors and links to related genomic, clinical, and proteomic information. Here, we describe updates to our resource, including improvements to our search facility and new download features.

## INTRODUCTION

The HUGO Gene Nomenclature Committee (HGNC) has been in operation for over 40 years and maintains one of the longest running databases that delivers biological standards for the scientific community. The HGNC is the sole authority for approving human gene symbols and corresponding descriptive gene names. HGNC is an Elixir UK service node (1) and a recommended resource on FAIRsharing (2). Standardised gene nomenclature is essential for effective scientific communication and facilitates retrieval of information about genes. The HGNC works with researchers, gene annotators and representatives from the nomenclature committees for other species to name protein-coding genes, pseudogenes and non-coding RNA genes. We record previous symbols and names, and alias symbols and names. We recently published an article ‘The risks of using unapproved gene symbols’ (3) that presents examples of where the use of alias symbols can cause problems, to raise awareness of this to the research community. We have also published a review of how we name long non-coding RNA (lncRNA) genes (4), which includes a description of how the seven most highly published lncRNA genes were named.

Genes with HGNC-approved nomenclature are all assigned a unique ID in the format HGNC:#, where # is a unique number. HGNC IDs are stable and do not change if the gene symbol changes. Therefore, we encourage all resources discussing/citing human genes on a large scale to use HGNC IDs. These IDs should also ideally be mentioned in the literature, especially when the symbol of one gene matches the alias of another to disambiguate between such genes, e.g. *GRID1* (HGNC:4575) has the symbol alias GluD1 and there is a separate gene with the approved gene symbol *GLUD1* (HGNC:4335).

**Figure 1.**
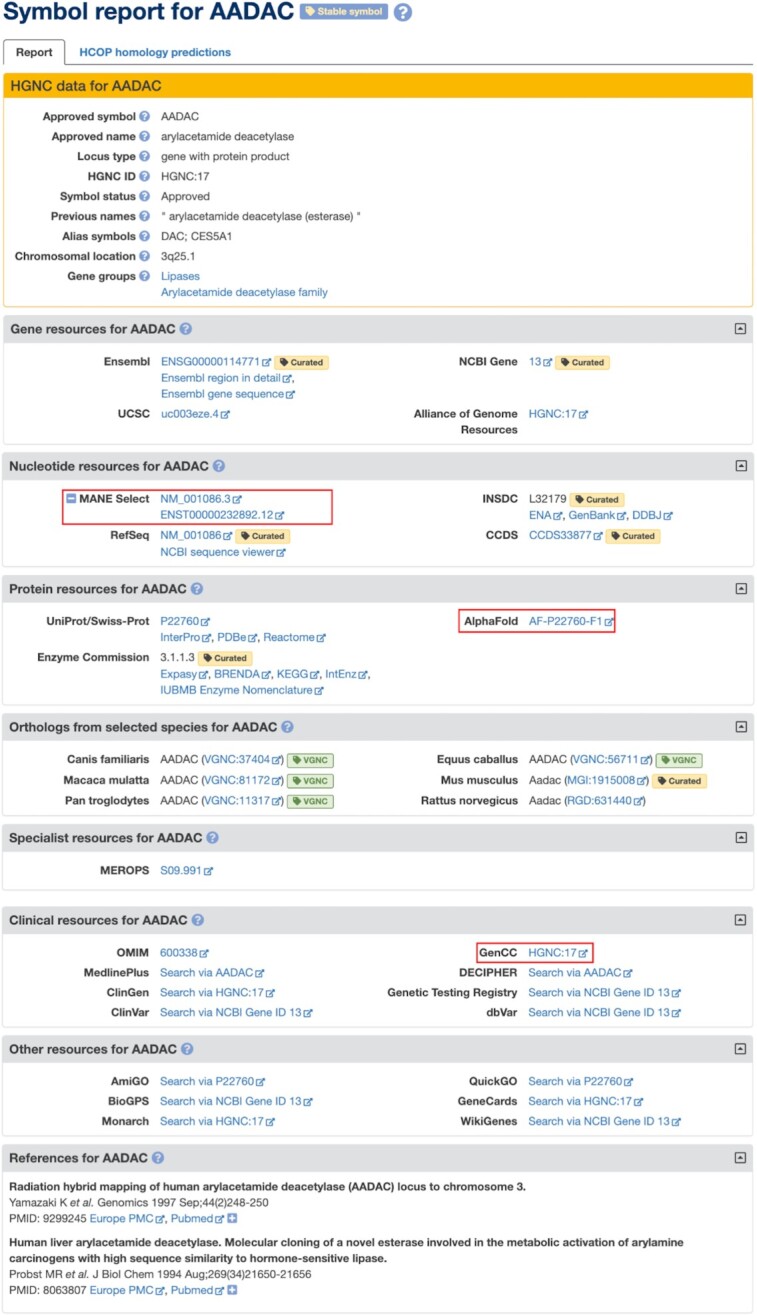
An example Symbol Report from www.genenames.org. New links to MANE Select, AlphaFold and GenCC are highlighted in red. The symbol *AADAC* has been marked by HGNC curators with the Stable symbol tag, meaning that the HGNC considers it unlikely that this symbol will ever need to change. The HGNC data box at the top of the Report presents HGNC-curated nomenclature, HGNC ID, chromosomal location and membership of gene groups. The rest of the Symbol Report displays links to biomedical resources, orthology resources and key references.

For each approved gene, the HGNC creates a Symbol Report on www.genenames.org that lists our approved nomenclature and related useful information (see Figure 1 for an example). The top section of the report entitled ‘HGNC data’ includes our approved nomenclature, locus type, HGNC ID, aliases, previous nomenclature where applicable, status (approved or withdrawn), and chromosomal location. In addition, the HGNC data section may include curator notes and/or links to gene groups curated by the HGNC. HGNC Symbol Reports also provide numerous useful external data links to biomedical resources such as NCBI Gene (5), Ensembl (6) UniProt (7), GeneCards (8), RNAcentral (9), UCSC genome browser (10), OMIM (11), ClinGen (12), GeneTests (13) and Monarch (14), which are sorted by type into the categories Gene Resources, Nucleotide Resources, Protein Resources, Clinical Resources and Other Resources. The ‘Orthologs from Selected Species’ section contains links for orthologs, if present, to MGD for mouse (15), RGD for rat (16) and VGNC (the HGNC’s sister project, the Vertebrate Gene Nomenclature Committee) for chimpanzee, rhesus macaque, dog, cat, horse, cattle and pig. For protein coding genes there is a separate ‘HCOP homology predictions’ tab that provides orthology predictions for 19 species, including all major model organisms, aggregated by our HGNC comparison of orthology predictions (HCOP) tool. The most recent version of the HCOP tool is described in full in (17).

In addition to HCOP, genenames.org has a general search function at the top of every page and a Multi-Symbol Checker tool that checks any inputted symbols against all HGNC approved symbols, previous, withdrawn and alias symbols in our database. We encourage authors to use this tool to check the gene nomenclature within their papers prior to submitting to journals. We provide several different options for downloading HGNC data, including prepared download files, a BioMart server, a Custom Downloads tool and a REST service. We also have a separate blog site (https://blog.genenames.org/) where we publish our regular newsletters, discussion points about gene nomenclature, guest posts from our collaborators and guides on how to use different aspects of our site.

The HGNC database is freely available to all via the web without the need to register or login and is accessible and legible on phone and tablet screens. All data is available for download in both TXT and JSON format.

Here we describe the changes made to the HGNC resource since our last report in 2021 (18). Note that the 2021 report included our VGNC resource; we will not describe this resource here but plan to publish a separate report in 2024 that will describe the VGNC site (https://vertebrate.genenames.org/) in full.

## HGNC DATA

### New gene entries in genenames.org

We have assigned approved nomenclature for 1028 previously unnamed genes within the last two years, including 870 new lncRNA gene entries and 136 new pseudogene entries. This brings our total count of gene entries to 43 170 as of 10 August 2022. We have increased the number of protein coding genes slightly from 19 211 to 19 229 since September 2020 because the naming of currently known protein-coding genes is essentially complete. The new additions include those added after discovery by research groups such as *H2BN1* (H2B.N variant histone 1; HGNC:56200) (19) and *LCE7A* (late cornified envelope 7A; HGNC:55921) (20) and those added by gene annotation groups but not yet studied elsewhere such as *AQP7B* (aquaporin 7B; HGNC:53895) and *CPHXL2 (*cytoplasmic polyadenylated homeobox like 2; HGNC:55919).

The majority of lncRNA genes were named using our systematic naming protocol (described in full in (21); 136 lncRNA genes were assigned the LINC (for long intergenic non-protein coding RNA) root symbol, e.g. *LINC02942* (HGNC:55957); 340 were named as antisense to protein-coding genes with the symbol suffix -AS e.g. *BICD1-AS1* (HGNC:55475); 346 were named as being divergent transcripts of (i.e. sharing a bidirectional promoter with) protein-coding genes with the symbol suffix -DT, e.g. *ACAD9-DT* (HGNC:56086); 12 were named as host genes for either microRNA or small nucleolar RNA genes, e.g. *MIR142HG* (HGNC:55980). Like protein-coding genes, lncRNA genes can be approved with unique symbols based on those published or suggested by research groups; examples of lncRNA genes named based on publications within the last 2 years include *CPMER* (cytoplasmic mesoderm regulator; HGNC:55992) (22) and *SCIRT* (stem cell inhibitory RNA transcript; HGNC:55341) (23).

Pseudogenes can be the result of duplicated genome sequence (also known as unprocessed pseudogenes), retrotransposed from mRNA (also known as processed pseudogenes) or can have degraded in situ. Where human pseudogenes have degraded in situ there are often species with functional copies at a conserved genomic location (also known as unitary pseudogenes). One such example that the HGNC named within the last 2 years is *FSIP2LP* (HGNC:55625); this was named relative to the VGNC protein coding orthologs *FSIP2L* (in horse (VGNC:109005), pig (VGNC:109145), cat (VGNC:109211) and dog (VGNC:109285)), and *Fsip2l* (MGI:2685441) in mouse. Examples of duplicated pseudogenes that we named include *GOLGA6EP* (HGNC:55708) and *GOLGA6FP* (HGNC:49206) that are present on a cluster with other GOLGA6 protein coding genes. Processed pseudogenes are usually named relative to their parent gene; recently named examples include *MTCH1P1* (HGNC:55886), *WEE1P1* (HGNC:56216) and *RACK1P2* (HGNC:55464).

### Marking genes as stable

As outlined in our current guidelines (24) ‘the stability of gene symbols, particularly those associated with disease, is now a key priority’. The HGNC has recently joined the Gene Curation Coalition (GenCC) (25), a project that brings together multiple groups that are either directly involved in gene-disease curation or promote standards needed to support this curation. The HGNC is contributing to this project by reviewing and evaluating the symbols of clinically relevant genes in the GenCC database. The review process primarily checks that approved symbols are not misleading and cannot be considered as pejorative or offensive. Secondary to this, curators consider approved symbol versus alias symbol usage, and whether the approved symbol causes problems for literature searching or data processing, although these factors are judged on a case-by-case basis and do not always trigger a symbol change. If changes are necessary, curators contact research groups, clinical groups and patient groups if available to discuss the suitability of proposed changes. Where curators conclude that symbols are extremely unlikely to ever need to be changed, the Symbol Reports are marked with our ‘Stable tag’ (as shown at the top of the *AADAC* Symbol Report in Figure 1). As of August 2022, the HGNC has applied the ‘Stable tag’ to 2964 genes, an increase of 48% since our previous database publication (18).

### Renaming pre-existing gene entries

Although the HGNC is committed to keeping gene symbols as stable as possible, we do make changes to symbols if necessary. When we make such changes the HGNC ID stays the same as shown in the examples below. Therefore, it is essential for databases to track current approved symbols using HGNC IDs. One of our current aims is to replace placeholder nomenclature with informative gene nomenclature once suitable information is available. Our largest group of placeholder symbols are of the format ‘C%orf#’ where % represents the chromosome that the gene is located on, orf stands for ‘open reading frame’ and # is an iterative number. We have updated 43 genes with this symbol format since our last report. Two of these updates were based on a collaboration between the HGNC and experts on dyneins and their assembly factors which has been published (26)—HGNC:25081 was updated from *C16orf71* to *DNAAF8* (dynein axonemal assembly factor 8) and HGNC:17721 was updated from *C20orf194* to *DNAAF9* (dynein axonemal assembly factor 9). Other examples of C%orf# renames based on publications are the renaming of HGNC:21702 from *C7orf26* to *INTS15* (integrator complex subunit 15) (27) and the renaming of HGNC:28628 from *C12orf45* to *NOPCHAP1* (NOP protein chaperone 1) (28). In some cases, C%orf# symbols are renamed following annotation updates that result in a change of the gene locus type away from protein coding, either to long non-coding RNA or to pseudogene, e.g. HGNC:33774 has been renamed from *C8orf86* to *LINC03042* (long intergenic non-protein coding RNA 3042) and HGNC:21620 has been renamed from *C6orf201* to *TEX56P* (testis expressed 56, pseudogene).

The FAM# root (for ‘family with sequence similarity’) is another class of placeholder symbols and is used to group together sets of paralogous genes for which no other information was known at the time of naming. We have renamed 27 genes with this root in the last 2 years, including the FAM189 root symbol where all members have been renamed together with the ENTREP# (endosomal transmembrane epsin interactor) root symbol (HGNC:24820 is now *ENTREP1* instead of *FAM189A2*, HGNC:29075 is now *ENTREP2* instead of *FAM189A1* and HGNC:1233 is now *ENTREP3* instead of *FAM189B*) based on data from a publication about HGNC:24820 (29) and further discussions with the research group to decide on appropriate nomenclature for all family members. Further examples include HGNC:24587 and HGNC:28593, which have been renamed from *FAM126B* and *FAM126B* to *HYCC1* (hyccin PI4KA lipid kinase complex subunit 1) and *HYCC2* (hyccin PI4KA lipid kinase complex subunit 2), and HGNC:33877 and HGNC:30701, previously *FAM155A* and *FAM155B*, which have been renamed as *NALF1* (NALCN channel auxiliary factor 1) and *NALF2* (NALCN channel auxiliary factor 2).

KIAA# symbols are our third placeholder symbol set and were approved for genes identified by the Kazusa cDNA sequencing project. We have updated the symbols for 5 KIAA#s since our last report, including HGNC:26953 and HGNC:28960, that were renamed together from *KIAA1109* and *KIAA0100* to *BLTP1* and *BLTP2* (for bridge-like lipid transfer protein family member 1 and 2).

### New gene groups

**Figure 2.**
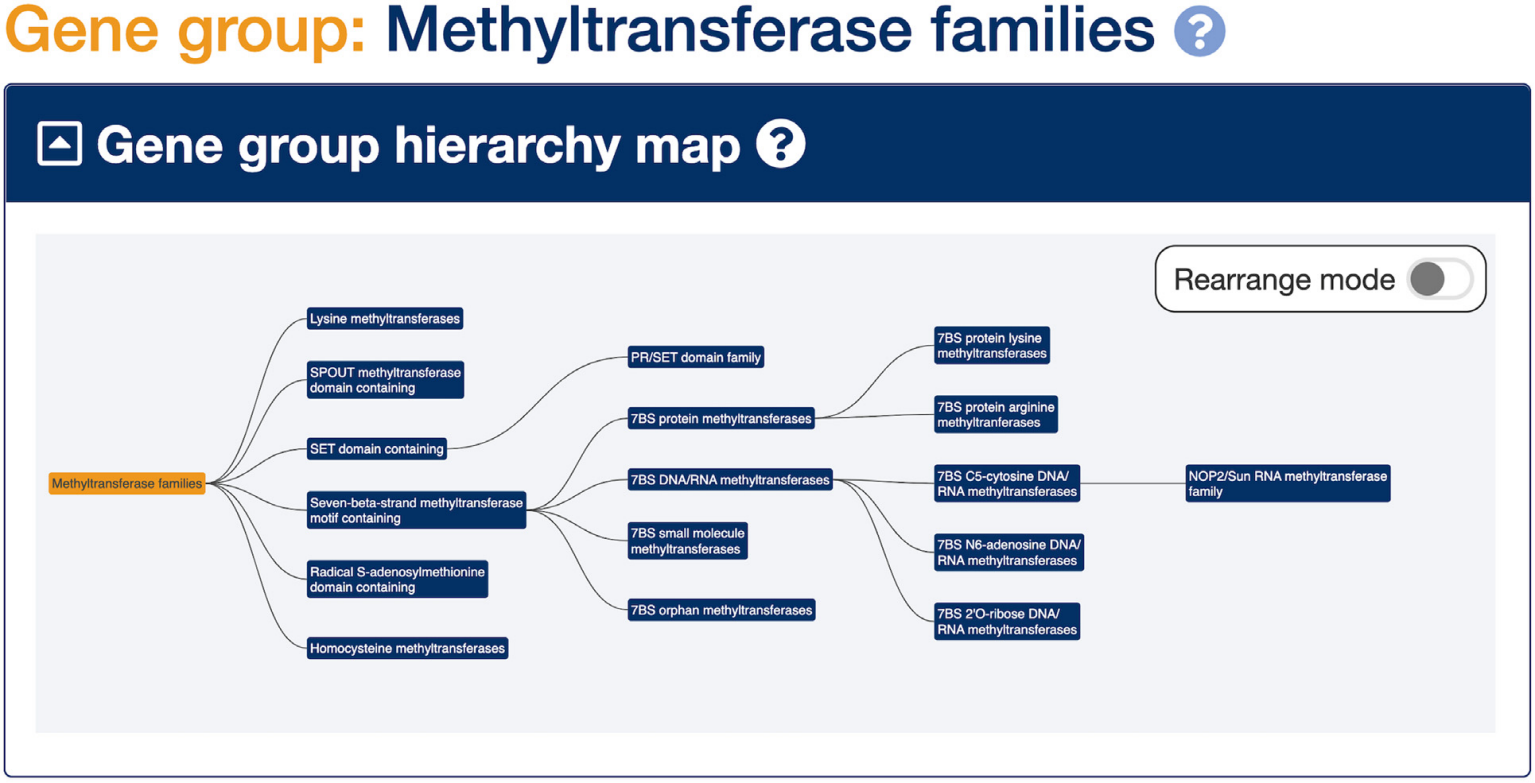
Gene group hierarchy for HGNC curated methyltransferase families. Users can navigate between gene groups by clicking on the gene group boxes.

The HGNC regularly names genes together with a common root symbol based on a shared characteristic, such as function, homology, or encoded protein structure/domains. We host gene group reports that represent both these shared root symbols and many other types of gene groupings such as membership of protein complexes, as well as gene families that are not named with a common root symbol. Since our last report we have added over 100 new manually curated gene groups to genenames.org. Examples of individual gene groups include the Transcription factor AP-2 family, PARN exonuclease family, Adducin family, CREC family and Mitochondrial translation release factor family. Related gene groups are curated into hierarchies that support browsing between groups. We have recently curated further methyltransferase gene groups, such as Homocysteine methyltransferases and Seven-beta-strand methyltransferase motif containing, and placed these into our Methyltransferases gene families group hierarchy (Figure 2) with help from our specialist advisor. We also worked on our Nuclear hormone receptors gene group hierarchy to make the subgroups consistent with the IUPHAR/BPS Guide to Pharmacology (30) nuclear hormone receptor pages.

## HGNC WEBSITE UPDATES

### Improved search facility

**Figure 3.**
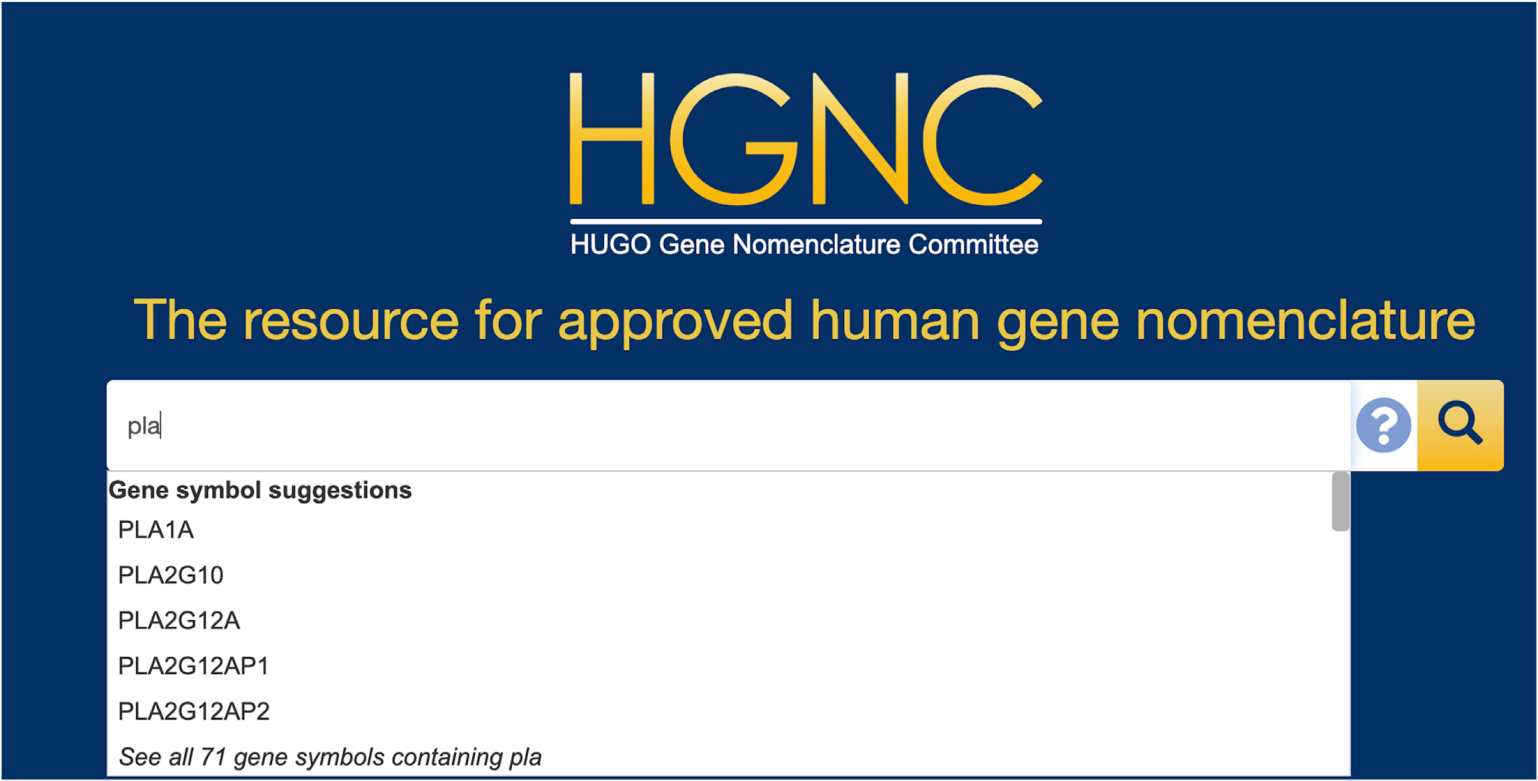
The new auto-suggest feature on the main search at www.genenames.org. The auto-suggest box is scrollable—this query also provides alias symbol suggestions, gene name suggestions and group name suggestions.

In April 2021, we launched a new, improved version of the search on genenames.org. This search now works across the entire website to include Symbol Reports, Gene group reports, announcements, articles and the separate blog.genenames.org site. One major improvement is the addition of an auto-suggest feature for several of our search categories, including approved symbols, previous symbols, aliases, gene names and group names. If there are many suggestions for a particular input term, the search auto-suggests five different matches for each category followed by a link that will show all results matching the term; for example, for the input ‘pla’ the search auto-suggests *PLA1A*, *PLA2G10*, *PLA2G12A*, *PLA2G12AP1* and *PLA2G12AP2* gene symbol suggestions, and then has the text ‘See all 71 gene symbols containing pla’ (Figure 3) which returns all of the results. The search no longer requires the inclusion of wildcards (*) so that the search term ‘cadherin’ now returns genes with this term in the gene name, alias name and previous name, and also returns the gene group page ‘Cadherins’. As many of our gene group names are plural, in the previous search there was a risk that they could be missed. The new search also accepts all major variant spellings between UK and American English, such as ‘signalling’ and ‘signaling’. The results of the search may now be downloaded in either TXT or JSON by clicking on the file icons in the ‘Download all results’ section below the search filters.

### New download features

Following several requests by our users, we now provide download files available in our HGNC archive (http://ftp.ebi.ac.uk/pub/databases/genenames/hgnc/archive/) in TXT or JSON format that show a list of symbol changes for the span of a month or a quarter of a year. The files contain the HGNC ID, approved symbol, previous symbol, gene name, locus type, and the date of the symbol change. The ‘symbol-changes’ files are available from 1 September 2021 onwards (the first file features changes made in August 2021) and accompany our ‘HGNC complete set’ and ‘withdrawn’ download files in the HGNC archive. Additionally, we have implemented a new REST API that will allow querying both HGNC and VGNC data.

Another new download feature is the provision of a Globus endpoint for HGNC data available from our Statistics and Download files webpage (https://www.genenames.org/download/statistics-and-files/). All HGNC download files can be downloaded from Globus. We recommend this method for users that need to download more than one file at a time, as it is rapid and is not affected by network glitches that may corrupt files during transfer.

### New links to other resources

For relevant genes we have added links to the GenCC project, described in the ‘Marking symbols as stable’ section above, to the Clinical Resources section of our Symbol Reports (Figure 1). And for all genes with a UniProtKB/Swiss-Prot (reviewed) protein link, we have also added links to view protein structures predicted by the Artificial Intelligence algorithm AlphaFold2 at the AlphaFold Protein Structure Database (31) from the Protein Resources section of our Symbol Reports (Figure 1). New links have also been provided from the Nucleotide Resources section of our Symbol Reports to MANE (Matched Annotation from NCBI and EMBL-EBI) Select transcript sequence records. The MANE project (32) aims to provide a set of standard transcripts for human protein-coding genes annotated by both the RefSeq and Ensembl/GENCODE projects, to aid in transcript variant reporting by the clinical and research community. MANE transcripts list versioned RefSeq and Ensembl IDs, i.e. our *AADAC* Symbol Report displays both the RefSeq ID NM_001086.3 and the Ensembl ID ENST00000232892.12 (Figure 1). GenCC, AlphaFold and MANE links are all available to download via our REST service.

## FUTURE PLANS

We will continue reviewing the nomenclature of clinically relevant genes and increase the number of Symbol Reports marked with the Stable tag. We will also continue to approve informative symbols and names for published long non-coding RNA genes and for newly identified human protein-coding genes, such as those that may be annotated in future based on Ribo-Seq data (33). We will continue to name annotated pseudogenes and long non-coding RNA genes following our systematic protocols. We will explore automating the naming of a subset of long non-coding RNA genes that follow a set of defined annotation rules. We will continue to update placeholder symbols of protein-coding genes with informative nomenclature whenever possible.

In addition to manually curating new gene groups, we plan to review our gene group resource, including how we connect gene groups within hierarchies, and will explore marking gene groups with types, such as ‘shared homology’ and ‘shared function’. For example, the *AADAC* Symbol Report in Figure 1 shows *AADAC* as a member of two different gene groups: ‘Lipases’, which is a functional grouping based on enzymatic activity, and ‘Arylacetamide deacetylase family’, which represents a group of paralogs. Applying types to these groups would be informative and would allow users to filter or search groups by type.

We will also continue to explore the creation of an InterMine service (34) for querying and downloading HGNC and VGNC data, which would have the advantage of being interoperable with other InterMine tools but would include more HGNC (and VGNC)-specific data than the currently available HumanMine, e.g. our stable human symbol set and gene group data would be incorporated.

## DATA AVAILABILITY

HGNC services are freely available at https://www.genenames.org/. HGNC code is available at the GitHub repository (https://github.com/HGNC).
